# Knowledge and understanding of information after taking decision to participate or not in a randomized trial of surgery versus radiotherapy among patients with locally advanced prostate cancer – an observational study

**DOI:** 10.2340/1651-226X.2025.42218

**Published:** 2025-01-28

**Authors:** Yvonne Brandberg, Olof Akre, Mia Bergenmar

**Affiliations:** a Department of Oncology-Pathology, Karolinska Institutet, Stockholm, Sweden; b Department of Urology, Karolinska University Hospital, Stockholm, Sweden; c Clinical Epidemiology Unit, Department of Medicine, Karolinska Institutet, Stockholm, Sweden; d Department of Care Science, Sophiahemmet University, Stockholm, Sweden

**Keywords:** Clinical trials, prostate cancer, quality of informed consent, EORTC QLQ-INFO25, satisfaction with information

## Abstract

**Background and purpose:**

Informed consent from trial participants is mandatory. In a randomized clinical trial, we investigated (1) differences in knowledge and understanding of trial information between patients who participated and those who refrained, (2) differences in perceptions of information, and (3) differences in satisfaction with the information.

**Patients:**

After the decision about participation in the randomized study, ‘Surgery versus radiotherapy for locally advanced prostate cancer’ (SPCG-15), patients were sent questionnaires (‘Quality of Informed Consent’, EORTC QLQ-INFO25). Patients were categorized in ‘Non-participants’ or ‘Participants’.

**Results and interpretation:**

A total of 80 patients (80%) responded, 68% of non-participants and 95% of participants. Between-group differences in knowledge were found for duration of the trial, insurances in the trial, and if the trial intervention had been proven to be superior. Patients had high levels of knowledge (> 80%) regarding the trial aim, that participation implied research, the right to decline, that future patients benefit from research and, of the randomization procedure. Less than 50% responded correctly concerning risks associated with the trial, the unproven nature of the trial and issues about insurances. Non-participants scored lower concerning duration of trial participation, confidentiality of medical records, treatments and procedures in the trial, and experimental nature of treatments. There were no differences regarding satisfaction with information.

Non-participants and participants did not differ in satisfaction, or in knowledge and understanding of most aspects of the information. Knowledge levels were low in some areas, and thus, it seems to be room for improvement to fulfill the requirements of informed consent.

## Introduction

Successfully completed clinical trials are paramount in developing treatments against cancer. The requirements regarding the potential participant’s knowledge and understanding are stated in the Helsinki Declaration [1]. Informed consent from potential clinical trial participants is mandatory. The information must present the aim of the study, the procedures included, and which ones are experimental, the duration of study, potential risks, discomforts and benefits, alternatives to participation, confidentiality, procedures in case of harm in connection with the study and who to contact regarding study-related issues [1].

In general, only about half of adult patients invited to clinical cancer trials choose to participate [2]. Low recruitment rates might have several negative clinical, scientific, economic, and ethical implications [3–5]. Knowledge about important factors in the recruitment process is essential for successfully recruiting and retaining study participants. More than 100 barriers, facilitators and other factors of importance for participation in clinical trials have been identified in an overview of reviews [6]. The factors are of diverse nature and relate to the clinical trial, potential participants, clinical trial information, and benefits and costs related to participation. Information-related factors concern ‘Need for information about the clinical trial’, that can act as both a barrier and facilitator. Misconceptions and misunderstandings are other factors related to clinical trial information, acting as barriers to participation [6]. In a previous study, we found associations with decision to participate and higher levels of knowledge and understanding related to the trial [7]. That study, however, included patients from various trials with a mix of cancer diagnoses and trial phases.

In the present study of patients with locally advanced prostate cancer invited to participate in a randomized prostate-cancer clinical trial, we aimed to investigate whether there are differences in knowledge and understanding of the trial information between patients who refrained from participation in that trial (non-participants) and those who decided to participate (participants). Moreover, we aimed to assess differences between non-participants and participants in their perception of information concerning the disease and its treatments as well as any between-group differences in satisfaction with the information.

## Patients and methods

### Patients

Newly diagnosed patients with locally advanced prostate cancer were informed about the seriousness of the disease and the need for treatment. The information also included an invitation to participate in an ongoing randomized study, ‘Surgery versus radiotherapy for locally advanced prostate cancer’ (SPCG-15) (Clinical trial gov nr: NTC02102477). The SPGC-15 recruited patients from the Nordic countries. The present study included patients from Karolinska University Hospital, Sweden. Radiotherapy was considered as the standard treatment and surgery as the experimental treatment. Trial information was provided orally by both a surgeon and an oncologist, in the same session or separately.

### Data collection

During the period from December 2015 to August 2021, the principal investigator (M.B.) received information about eligible patients (name and address) from a research nurse responsible for monitoring the trial at the Clinical Trial Unit, Karolinska University Hospital. Within 2 weeks after having decided whether to participate or not, consecutive patients were sent an information letter about the present study at Karolinska Institutet by regular mail from the study coordinators (M.B. and Y.B.). None of them were involved in the clinical trial. In addition, we enclosed questionnaires on knowledge and understanding of the trial information and patients’ perception of received information, together with a prepaid return envelope. We sent reminders to those who did not respond within 2 weeks. Returned completed questionnaires were considered as patient consent to participate. The patients were categorized according to whether they have decided to participate or not in the clinical trial SPCG-15 in two categories, ‘Non-participants’ or ‘Participants’.

### Questionnaires

The questionnaire ‘Quality of Informed Consent’ [8, 9] consists of two parts. Part A includes 20 items measuring patients’ knowledge of crucial aspects of clinical trials. The responses are given in three categories: ‘Disagree’, ‘Unsure’, ‘Agree’. Part B consists of 14 items, where the patients are asked to indicate to what extent they understood the different aspects of the trial. The responses are given in five categories from ‘I didn’t understand this at all’ (1) to ‘I understood this very well’ (5). The Swedish translations followed the guidelines from the European Organization for Research and Treatment of Cancer (EORTC) Quality of Life Group and were pilot tested on 16 patients [10]. The items were adapted to whether the patients had consented to participate or not. For Non-participants, some items ended with ‘…the trial you were invited to participate in’. For Participants, the same items ended with ‘…the trial you consented to participate in’.

We selected the items in the section concerning the informed consent procedure (e.g. time spent, present persons), additional sources of information, previous trial participation, difficulty making decision about trial participation and education from the Quality of Informed Consent questionnaire, Part C [9].

The European Organization for Research and Treatment of Cancer Information Module (EORTC QLQ-INFO25) includes 25 items concerning patients’ perception of the information received during the disease and treatment period [11]. Four subscales regarding the disease, medical tests, treatments and other services are derived from the items. In addition, eight single items are analysed, including satisfaction with information. For 21 of the items, the response format ranges from ‘Not at all’ (1) to ‘Very much’ (4). The remaining four items are responded to with ‘Yes’ or ‘No’. In a review of instruments for the assessment of patients’ information needs, the EORTC QLQ-INFO25 was the only questionnaire found, among 21, to have had psychometric property testing guided by established guidelines [12]. In addition, the authors appreciated the rigorous development process, also including patients.

### Statistical analyses

When scoring Quality of Informed consent Part A, each correct response to the items was assigned a score of 100 and incorrect answers and responses in the category ‘Unsure’ were assigned a score of 0 according to one of our previous papers [7]. Scores for the items in Informed consent Part B ranged from 1 (‘Did not understand this at all’) to 5 (‘I understood this very well’). Mean values for each item were calculated. Number of sources of information were summarised (items 6 to 9, Part C). EORTC QLQ-INFO25 was scored according to the original paper [11]. Mean scores were transformed to 0 to 100 points scales.

We compared the proportions of correct responses between non-participants and participants using Chi^2^-tests for categorical variables, and students’ *t*-test for continuous variables. The level of statistical significance was set to *p* ≤ 0.01, to minimize the risk of multiplicity.

The study was approved by the Regional Ethics Committee, dnr: 2015/2059-32.

## Results

**Figure 1:**
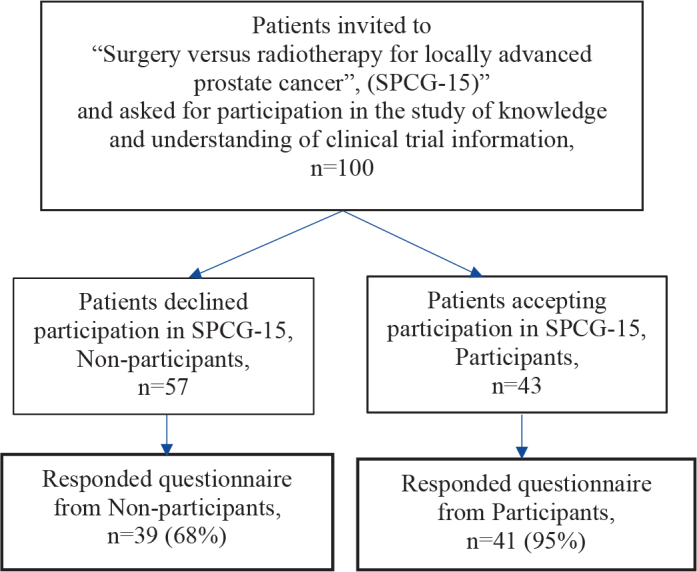
Flow chart for inclusion.

A total of 100, 57 (57%) non-participants and 43 (43%) participants, were invited to participate in the present study. A total of 80 patients (80%) responded to the Information study questionnaires. They are presented by participation status (‘Non-participants’ or ‘Participants’) in the flow chart for inclusion (Figure 1). The mean age was 68 years among non-participants and 69 years among participants.

### Knowledge of trial information (Quality of Informed Consent Part A)

**Figure 2:**
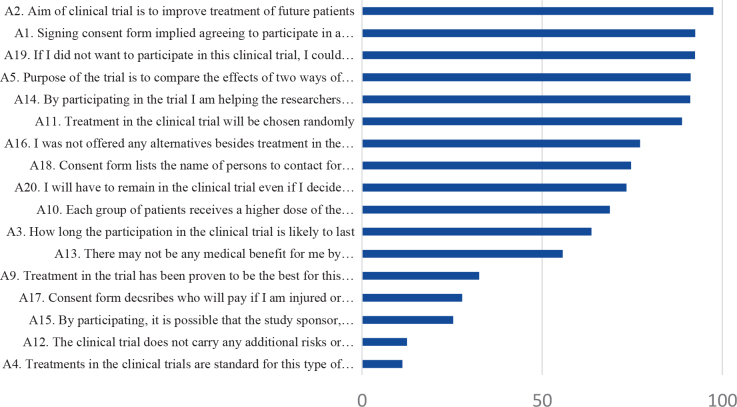
Proportion of correct responses to the items asking about knowledge concerning trial information for the total sample.

The proportion of correct responses to the items asking about knowledge concerning trial information is presented for the total sample in Figure 2. Overall, high levels of knowledge (> 80%) were found for six of the items. These items concerned knowledge about that the aim of the trial was to improve treatment for future patients, that signing the informed consent implies participation in research, the possibility to decline participation, that the purpose of the study was to compare two treatments, that participation was useful for future patients and knowledge about randomization. Less than 50% responded correctly to five of the items. These items concerned risks associated with the trial, the unproven nature of the trial and issues about insurances in connection to participating in the trial. Statistically significant differences in knowledge between non-participants and participants were found for three items. A lower proportion of non-participants responded correctly concerning the duration of participation in the clinical trial and about insurances related to the trial. In addition, a higher proportion of non-participants responded correctly on the item asking whether any of the treatment arms in the trial had been proven to be the best.

### Understanding of trial information (Quality of Informed Consent Part B)

**Figure 3:**
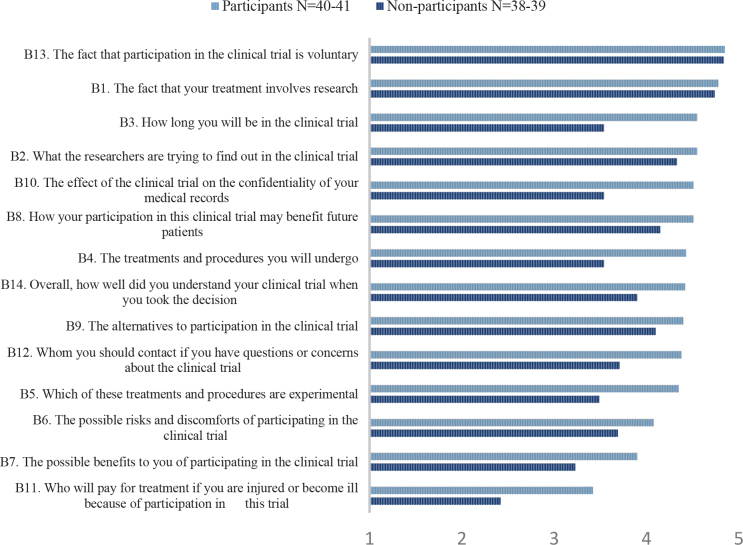
Mean scores on the items asking about perceived understanding of trial information according to participants and non-participants.

Mean scores on the items asking about understanding of trial information are presented for the two groups separately in Figure 3. Overall, the non-participants scored lower than the participants on all items regarding understanding, with statistical significant differences for four items. These four items concerned duration of participation in the trial, confidentiality of medical records, the treatments and procedures in the trial, and the experimental nature of the treatments and procedures.

### The informed consent procedure (Quality of Informed Consent Part C)

**Figure 4:**
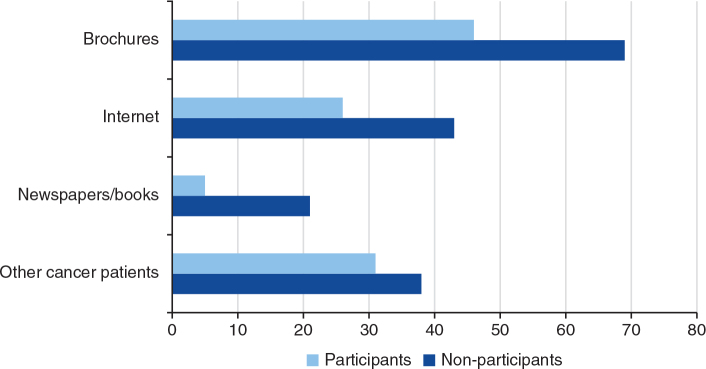
Proportions using various information sources during the consent procedure according to participants and non-participants.

The proportion of patients’ use of various information sources during the consent procedure, according to participation or not, is presented in Figure 4. No statistically significant differences were found between the groups regarding use of the various sources.

There were no statistically significant between-group differences regarding the items asking about presence of a family member/friend or nurse during the discussion about participation in the trial with the physician (data not shown). Neither were there any differences found in the duration of the consultation. When asked about whether it was easy or difficult to decide on participation in the trial, a statistically significant difference was found. A total of 25% of the non-participants reported the decision to be ‘Difficult’ or ‘Very difficult’ to make. The corresponding figure for participants was 10% (*p* = 0.01).

### Comparisons in perceived information concerning the disease and its treatments between the groups (EORTC QLQ-INFO25)

In Table 1, mean values, standard deviations, and *p*-values for between-group comparisons on subscales and single items for the EORTC QLQ-INFO25 are presented for the two groups separately. Three items had ‘Yes’ or ‘No’ as response alternatives presented below (data not presented in Table 1). There were no statistically significant differences between the groups on any of the subscales or items, including satisfaction with information. Out of the non-participants, 32 (86%) reported that they had received written information, but 5 (14%) responded that they had not. Corresponding figures for participants were 24 (69%) and 11 (31%). More information was requested by 51 (51%) of the non-participants and by 67 (48%) of the participants. The item asking about if the patient wished for less information was responded to by all patients by indicating ‘No’.

**Table 1:** Mean scores, standard deviation and *p*-values for subscales and single items in EORTC QLQ-INFO25.

Subscales and single items	Participants (*n* = 35–38)	Non-participants (*n* = 36–37)	*p*
Information about:	Mean (SD)	Mean (SD)	
The disease	63 (16)	57 (20)	0.178
Medical tests	80 (19)	74 (23)	0.226
Treatments	77 (18)	73 (20)	0.385
Other services	31 (29)	32 (31)	0.914
Different places of care	31 (32)	30 (31)	0.903
Things you can do to help yourself	39 (34)	37 (38)	0.829
Satisfaction with information	75 (21)	68 (27)	0.203
Overall helpful information	75 (25)	79 (24)	0.503

SD: standard deviation.

Scale scores range from 0 to 100. High values indicate high levels of perceived information.

## Discussion

The present paper compared knowledge, understanding and perception of the trial information between men who chose not to participate in a prostate-cancer clinical trial, and men who choose to participate. They responded to questionnaires shortly after their decision about participation.

We found few differences between non-participants and participants regarding knowledge about the trial they were invited to participate in. Knowledge regarding the elements stated to be mandatory in trial information varied in the total sample, implicating some room for improvement of the information. Over 80% responded correctly concerning the trial aims, the consequences of signing the consent form, the possibility to decline participation, the concept of randomization, and the potential benefits of participation for the future care of patients with prostate cancer. Less than 40%, however, knew the right answers to the items stating that the treatment in the trial have been proven to be the best and that the treatments in the trial are standard. Similar results for these two items were found in our previous studies [10, 13]. The item about additional risks and discomfort associated with the trial was correctly responded to by only 12% of the study population. It is of great importance that patients have knowledge about the fact that one treatment condition is experimental. ‘Therapeutic misconception’ might also have been present. This phenomenon is characterized by difficulty to distinguish between research and treatment and is associated with overestimation of benefits and underestimation of risks [14]. In addition, risk information might not be emphasized by the physicians including patients [6].

In general, non-participants reported lower levels of understanding than participants concerning duration of the trial, the effect of participation on the confidentiality of the medical records, the treatments and procedures in the trial, and which of them that were experimental. One explanation for these differences is that the participants’ higher experienced understanding contributed to that they actually took the decision to participate. It is also possible that they overestimated their understanding since they decided to participate. These results are in concordance with the findings in one of our previous studies in which non-participants scored lower on both knowledge and perceived understanding [7].

We found that a higher proportion of non-participants thought that the decision was difficult to take. Not understanding the information may have been one reason for the experienced difficulty. Another explanation, contributing to making the decision to abstain difficult, is that the patients might have felt that the physician informing them about the trial wished that they would consent to participate. Thus, taking the decision not to participate might have felt more demanding. Participants, on the other hand, might have followed what they experienced as their physicians’ recommendation according to previous research, showing that the patient-physician relation was an important factor for participation in clinical trials [15–17].

No differences were found between the groups for the items regarding the informed consent procedure. Thus, neither presence of a relative/friend or nurse, nor the duration of the consultation did show any associations with consenting to participate in the trial. These findings are in concordance with one of our previous studies [18].

The patients were mostly satisfied with the information received and found it helpful. About 50% of the patients in both groups requested, however, further information. No patient wished for less information. The patients reported having received most information about the disease, medical tests and treatments. In a review of 104 studies of patients’ information needs, treatment-related information comprised the largest proportion of unique information needs during the diagnosis and treatment phase [19]. Patients in the present trial reported less information concerning ‘Other services’, ‘Different places of care’ and ‘Things you can do yourself’. Considering the fact that the patients received the information and took the decision soon after diagnosis and before start of treatment, it is plausible that the focus of the information was on the treatment.

The use of validated questionnaires and that consecutive patients were invited are strengths of the study. In addition, the patients were recruited from one randomized trial. One limitation is the fact that the information provided to the patients was not recorded. Thus, we lack knowledge of how the information actually was provided. The relatively small sample size decreases the sensitivity to detect differences between the comparison groups.

Knowledge and perceived understanding showed few differences between non-participants and participants. Levels of knowledge in both groups were low for items regarding the experimental nature of the trial and about the risks involved. Non-participants found the decision to abstain from the trial more difficult to take. Although knowledge did not show associations with participation in the trial, there appears to still be room for improvement in order to fulfill the requirements for informed consent.

## Data Availability

Data is available on request from the corresponding author.
